# Heat Stress: A Hazardous Occupational Risk for Vulnerable Workers

**DOI:** 10.1016/j.ekir.2023.05.024

**Published:** 2023-06-01

**Authors:** Priyadarshini John, Vivekanand Jha

**Affiliations:** 1 AIG Hospitals, Hyderabad, Telangana, India; 2 George Institute for Global Health, New Delhi, India; 3 School of Public Health, Department of Medicine, Imperial College, London, UK; 4 University of New South Wales, Sydney, Australia; 5 Prasanna School of Public Health, Manipal Academy of Higher Education, Manipal, India

See Clinical Research on Page 1363

Anthropogenic climate change has caused extreme heat events to become more frequent, intense, and long lasting. Prolonged exposure to extreme heat overwhelms the body’s ability to thermoregulate, leading to heat-related illnesses and exacerbating preexisting health conditions.^1^ Heat stress causes more deaths than any other weather-related cause worldwide. Recurrent exposure to extreme heat is associated with the development of new-onset cardiovascular, kidney, and lung disease, and has devastating and wide-ranging effects on mental health and wellbeing, including worse mental fatigue, higher aggression, and increased suicide rates. This connection is not limited to extreme heat events, but also affects people living in consistently hot climates.^2^

Nearly 40% of the global population is exposed to >30 ^°^C ambient daytime temperatures all year round.^3^ The mean temperature in India has risen by 0.7 °C between 1901 and 2018 and is projected to rise by 4.4 ^°^C by the end of the century. The absolute number and the proportion of the world’s population at risk of adverse health effects from extreme heat is growing, with India being one of the most affected.^4^ According to the Lancet Countdown, heat-related deaths increased by 55% between 2000-2004 and 2017-2021, and 167.2 billion potential labor hours were lost because of heat exposure in India alone.^5^ On the basis of current projections, humid heat in India will approach 31 °C wet-bulb globe temperature (WBGT) by 2060 and breach 35 °C (the upper limit of survivability) by 2100.^6^ The impact of increasing temperatures as a result of climate change on the health of vulnerable populations who work outdoors for long hours cannot be overstated. Heat is an occupational health hazard for workers in various industries, including agriculture, construction, and transportation, who are exposed to high temperatures for prolonged periods, often without adequate access to adaptation strategies such as shade, rehydration, or rest breaks. They may also be hesitant to report symptoms of heat stress because of fear of job loss or retaliation. In addition, many of these workers are undocumented and do not have access to healthcare, further exacerbating the risk of heat-related illness.

Much of the literature on occupational heat stress is derived from agricultural areas. The current issue of the journal presents a study by Venugopal *et al.*,^7^ aimed at computing the effects of heat stress in a nonagricultural environment.^7^ This cross-sectional study was conducted over 3 years among 352 adult workers (median age 47 years) from 7 salt pans in Tamil Nadu, India (Figure 1). Salt pans are flat sheets of ground covered with salt and other minerals formed in rivers and ponds where the rate of evaporation is greater than precipitation. Studying heat stress and its effects in nonagricultural locations eliminates probable confounding factors, such as pesticides and other toxins implicated in disease causation and helps to study the direct link between disease and heat stress.

**Figure 1:**
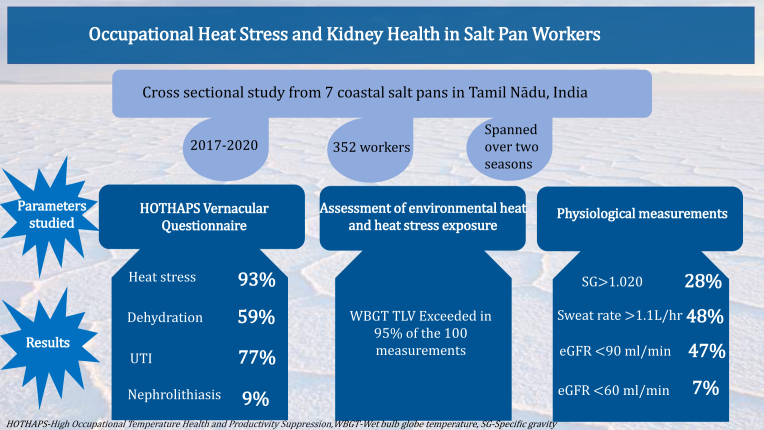
Subjective and objective assessment of kidney function in coastal salt pan workers from India. eGFR, estimated glomerular filtration rate; TLV, threshold limit value; UTI, urinary tract infection; WBGT, wet-bulb globe temperature.

The authors used the Heat Observation and Technical Health Assessment of Participants by Satellites questionnaire, adapted for local circumstances, to measure the individual level of heat stress. This tool was developed by the World Health Organization to assess the risk of heat stress in outdoor workers and consists of questions that assess the physical demands of the job, the level of exposure to heat, and the presence of heat-related symptoms. They also used WBGT, validated and recognized for measuring heat stress in various occupational and sports settings to monitor area-level heat stress. WBGT is a composite measure of environmental factors that affect human thermal comfort and physiological responses that contribute to heat stress, including air temperature, humidity, air movement, and sun angle. WBGT was measured at 100 locations over 19 days in both summer and winter, with repeated measurements throughout a shift. Jobs were categorized as moderate (15%) or heavy (85%). WBGT threshold limit values are 27.5 °C and 28 °C, respectively for heavy and moderate workload, as per the American Conference of Governmental Industrial Hygienists 2018 guidelines. Physiological measurements included measuring core body temperature, urine pH, proteinuria, hematuria and leukocytes, urine specific gravity, sweat rate, heart rate, and serum creatinine.

WBGT rose steadily during the day, surpassing the threshold limit value even for moderate work as early as 7:30 am in the summer months and reaching a peak by 1:30 pm. The WBGT exceeded the threshold limits in 95% of the 100 measurements recorded. Most heavy-load workers had their WBGT exceed the recommended threshold limit value. A total of 93% of the workers reported symptoms of heat strain, and 59% had symptoms of dehydration. Approximately 77% of the workers reported symptoms interpreted as urinary tract infection. These symptoms could have been due to extreme urinary concentration due to excessive sweating and notably low water consumption (<1 L for the entire shift). Of particular note was the demand placed on women workers by the lack of restroom facilities, which could have forced reduced water consumption before and during the shift to avoid using the restrooms.

Glomerular filtration rate (GFR) was determined only once and showed estimated GFR <60 ml/min per 1.73 m^2^ in 7% of workers. This rate is comparable to the prevalence of low estimated GFR in urban populations in Tamil Nadu.^8^ However, the GFR estimation equations overestimate true GFR in the Indian population. Therefore, the true GFR is likely to be lower in a greater proportion of workers.

The major strengths of the study are the extensive WBGT monitoring adjusted with threshold limit value, and season and physiological monitoring (core temperature, urine specific gravity, and sweat and heart rates). The Heat Observation and Technical Health Assessment of Participants by Satellites questionnaire gave a sense of heat-related symptoms by measuring the physical demands of the job and the level of exposure to heat. It is simple and easy to use and has been shown to have good reliability and validity in a range of settings. The tool, however, is limited by its self-reporting requirement and inability to account for individual factors that may increase the risk of heat stress, such as age, preexisting medical conditions, or medication use, which may underestimate the risk of heat stress in some individuals.

A December 2022 World Bank report projects that up to 75% of India's workforce, or 380 million people, depend on heat-exposed labor. The impact of heat on the kidneys as an occupational hazard is of particular concern. Heat stress increases hospital admissions because of a range of indications, including acute kidney injury, kidney stones, chronic kidney disease, and urinary tract infections.^9^ The current study is limited by its cross-sectional design and lack of paired pre-post monitoring of kidney function. Ideally, the tests should be repeated in the same set of workers to measure the acute and chronic effects of heat stress. In addition, the risk of heat-related illness is compounded by the interaction of heat with other kidney disease risk factors. For example, workers who are already at risk of kidney disease because of factors such as hypertension or diabetes (excluded in this study) may be more susceptible to heat-related kidney injury even when it does not cross the safety threshold for otherwise healthy individuals. Many of these people undertake multiple jobs and may be at risk of other occupational hazards, such as heavy metals or pesticides. The authors do not clarify whether this was the case in these workers.

Are countries at risk ready to face the climate change challenge and develop resilience? Adapting to minimize the risks of heat-related illness for vulnerable workers requires a multifaceted approach (Figure 2). Employers must ensure that workers have access to shade, water, and rest breaks; and should provide training on the symptoms of heat stress and the importance of reporting symptoms early. In addition, healthcare providers should be aware of the increased risk of heat-related kidney injury in vulnerable populations. They should provide education on the importance of staying hydrated and avoiding prolonged exposure to high temperatures.

**Figure 2:**
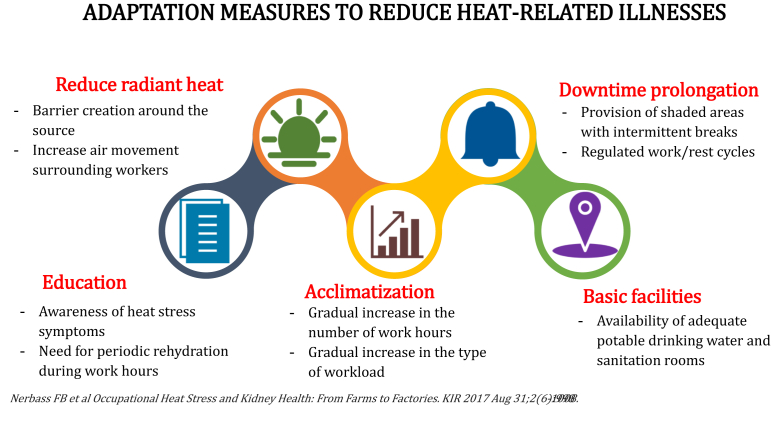
Adaptation strategies to decrease the incidence of heat stress hazards.

Government agencies can play a role in mitigating the risks of heat-related illness for vulnerable workers. The National Disaster Management Authority of India has developed guidelines for individuals to protect themselves from heat-related illnesses.^9^ These include staying hydrated, wearing loose-fitting and light-colored clothing, avoiding direct sunlight, taking breaks in cool or shaded areas, and avoiding alcohol, caffeine, and sugary drinks. There are recommendations for health care professionals, emergency responders, and community organizations, including setting up cooling centers in public spaces.

These guidelines are supplemented by the National Action Plan on Climate Change, which includes specific measures for improving public health infrastructure, increasing public awareness of heat-related illnesses, and strengthening the capacity of health care workers to diagnose and treat heat-related illnesses. However, these laws and regulations are good only if implemented. Agencies need to enforce labor laws that protect the rights of vulnerable workers, such as the right to breaks and access to healthcare.

In summary, as geographies worldwide experience increasing days of hot weather, heat stress becomes a growing occupational health risk, particularly for workers who spend extended periods outdoors or in hot environments. Lack of access to remedial measures and appropriate healthcare doubles the jeopardy. Employers, policymakers, and public health officials must prioritize the implementation of adaptation measures such as heat safety plans, shade structures, rest breaks, and access to cool drinking water to protect workers’ health and safety in a warming world.

## Disclosure

VJ has received grant funding from GSK, Baxter Healthcare, and Biocon and honoraria from Bayer, AstraZeneca, Boeringer Ingelheim, NephroPlus, and Zydus Cadilla, under the policy of all payments to the organization. PJ declared no competing interests.
